# Effects of Virtual Reality in the Rehabilitation of Parkinson’s Disease: A Systematic Review

**DOI:** 10.3390/jcm12154896

**Published:** 2023-07-26

**Authors:** Juan Rodríguez-Mansilla, Celia Bedmar-Vargas, Elisa María Garrido-Ardila, Silvia Teresa Torres-Piles, Blanca González-Sánchez, María Trinidad Rodríguez-Domínguez, María Valle Ramírez-Durán, María Jiménez-Palomares

**Affiliations:** 1 ADOLOR Research Group, Department of Medical-Surgical Therapy, Medicine Faculty and Health Sciences, University of Extremadura, 06006 Badajoz, Spain; jrodman@unex.es (J.R.-M.); mariajp@unex.es (M.J.-P.); 2 Bedmar Physiotherapy, Health and Movement Centre, 31006 Pamplona, Spain; 3 Research Group in Immunophysiology, Department of Medical-Surgical Therapy, Faculty of Medicine and Health Sciences, University of Extremadura, 06006 Badajoz, Spain; storres@unex.es; 4 Robolab Research Group, Medical and Surgical Therapy Department, Nursing and Occupational Therapy Faculty, University of Extremadura, 10003 Cáceres, Spain; 5 Department of Nursing, University Centre of Plasencia, University of Extremadura, 10600 Plasencia, Spain; valleramirez@unex.es

**Keywords:** Parkinson’s disease, gait, balance, virtual reality

## Abstract

Background: Parkinson’s disease is characterised by the loss of balance and the presence of walking difficulties. The inclusion of rehabilitation therapies to complement pharmacological therapy allows for comprehensive management of the disease. In recent years, virtual reality has been gaining importance in the treatment of neurological diseases and their associated symptoms. Therefore, the objective of this systematic review was to analyse the effectiveness of virtual reality on balance and gait in patients with Parkinson’s disease. Methods: This study is a systematic review conducted following PRISMA’s statements. An electronic search of the literature was carried out in the following databases: PubMed, Cochrane, Dialnet, Scopus, Web of Science, PsycINFO and Science Direct PEDro. The inclusion criteria were controlled and non-controlled clinical trials published in the last 12 years in English or Spanish, in which virtual reality was applied to treat balance and gait impairments in patients with Parkinson’s disease. Results: 20 studies were finally included in this review. A total of 480 patients participated in the included studies. All patients were diagnosed with Parkinson’s disease. Most of the investigations used the Nintendo Wii + Balance Board or the Microsoft Kinect TM combined with the Kinect Adventures games as a virtual reality device. Conclusions: According to the results of this literature review, virtual reality-based interventions achieve good adherence to treatment, bring innovation and motivation to rehabilitation, and provide feedback as well as cognitive and sensory stimulation in patients with Parkinson’s disease. Therefore, virtual reality can be considered an alternative for personalised rehabilitation and for home treatment.

## 1. Introduction

Parkinson’s disease is the most common neurodegenerative movement disorder [1]. This condition is characterised by the presence of motor and non-motor symptoms which are related to the damage of multiple structures of the central and peripheral nervous system [2,3,4]. These symptoms have a negative impact on coordination and on mobility [5], which has a significantly negative effect on the person and impairs the patient’s quality of life [5].

The most commonly used pharmacological treatment is the intake of Levodopa or an oral dopamine precursor [4,5,6,7]. Despite the current medical and pharmacological treatments, patients continue to progressively develop motor and non-motor impairments, such as sleep disturbances, cognitive impairment, and mood disorders [1]. This progression of the condition makes a comprehensive rehabilitation treatment essential, with the physiotherapist as part of the multidisciplinary team [8,9]. Physiotherapy in people with Parkinson’s disease will focus on six specific areas: transfers; posture; balance (falls); upper limb function; gait; and physical capacity and activity [10,11].

Currently, thanks to research and therapeutic and technological innovations, there are other treatments that can be complementary to pharmacology and conventional therapy. These include those treatment approaches based on the use of neurorehabilitation programmes by means of electronic systems, which allow rehabilitation to be extended beyond the health centre. Moreover, virtual reality is an innovative approach that has been gaining importance in the treatment of neurological diseases in both motor and non-motor impairments in recent years [12].

The term virtual reality was first used by J. Lamier in 1986. Although the definition of this term has changed over time, one of the most widely accepted is ‘the simulation of a real environment generated by a computer, in which a human–machine interface allows the user to interact with certain elements of the simulated scenario’ [13,14,15,16,17].

Virtual reality allows a therapeutic intervention based on the use of technologies with an interactive interface that recreates in real time the representation of a perceptual reality generated by the computer, with the patient being able to act and participate in this virtual environment [12]. It is important to note that virtual reality is a technology that allows the input and output of information in the system. In addition, the motor performance is displayed in the virtual environment, and subsequently, the system provides multimodal feedback related to the execution of the movement. Through the external and internal senses (proprioception), the sensory feedback is integrated into the patient’s mental representation [18,19]. The sensory feedback associated with the exercises in the virtual environment appears to activate the mirror neuron systems, which would be able to store in primary motor cortical areas a memory of the representation of the movement to be performed [20,21].

In addition, scientific research has evidenced that computational neuroscience, i.e., based on the use of computers and technologies, has demonstrated that the application of virtual reality offers a greater feedback service of the actions performed [22,23]. Likewise, this feedback allows greater improvements in motor learning and task performance compared to traditional training [23]. Both immersive and non-immersive virtual reality are currently used, although the coupling between perception and action in non-immersive virtual reality can be quite different than in the real world, which is why immersive virtual reality is used to achieve greater reality as patients may ‘forget’ that they are in a training situation [22,24,25].

On the other hand, exergaming programs based on entertainment platforms such as Nintendo Wii or adaptations thereof have been feasible for therapeutic use, improving abilities such as balance or quality of life and achieving high levels of satisfaction and adherence in people with Parkinson’s disease [26].

Based on all this, the objective of this systematic review was to know the effectiveness of virtual reality on balance and gait in patients with Parkinson’s disease.

## 2. Materials and Methods

### 2.1. Study Design

This systematic review was carried out following the PRISMA statement [27]. The review protocol is available in PROSPERO (registration number: CRD42021256172).

In order to identify relevant studies, the search was conducted in the following databases: PubMed; Cochrane; Dialnet; Scopus; Web of Science; PsycINFO; and Science Direct PEDro (Physiotherapy Evidence Database).

### 2.2. Search Strategy

The keywords used in the abstract and title fields were as follows: Parkinson’s disease; virtual reality; gait; balance; Parkinson’s disease; physical therapy; physiotherapy. These keywords were introduced in Spanish when the database required it. The Spanish terms used were as follows: Parkinson; realidad virtual; equilibrio; deambulación (Parkinson’s disease; virtual reality; balance; mobility). The keywords were combined with the Boolean operators AND or OR. The syntaxes of combined descriptors in the scientific database search can be found in Table 1.

### 2.3. Inclusion and Exclusion Criteria

The inclusion criteria were as follows: (a) both controlled (C) and non-controlled (NC) clinical trials; (b) published within the last 12 years; (c) in English or Spanish; (d) individuals ≥ 65 years. The search was limited to the last 12 years in order to analyse the most recent advances in the use of virtual reality in the variables under study and to update the scientific evidence available in the literature on this topic [28,29]. The exclusion criteria were established following the PICO model (population, intervention, control, comparison, and outcomes). Exclusion criteria were established as follows: the literature reviews or any type of document that is not a clinical trial; the use of treatment techniques that are not based on virtual reality; and treatments carried out on patients under 65 years of age.

### 2.4. Study Selection

A pre-selection of papers was performed, considering that they were within the proposed subject of the study. This selection was carried out by reading the abstract of the studies and excluding those that did not meet the established criteria.

**Figure 1:**
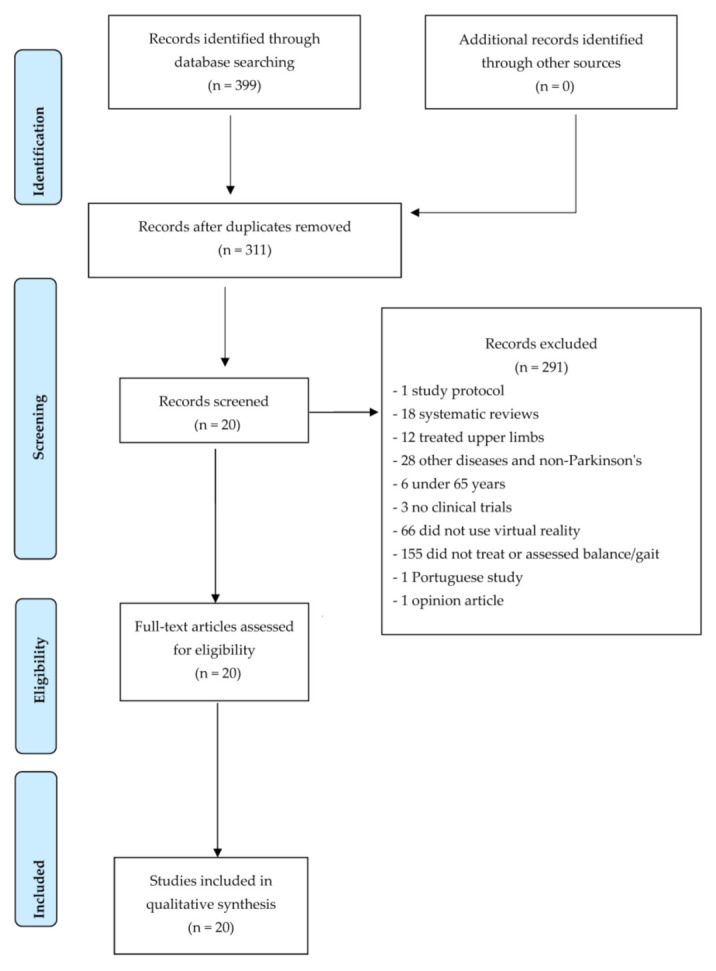
PRISMA flowchart.

The full text of the studies that met the inclusion criteria was revised, analysed, and included in the systematic review. All potential full-text articles were retrieved and evaluated by the two reviewers independently. Although the level of agreement between the two reviewers was not specifically calculated, any disagreements on the inclusion/exclusion of full-text articles were resolved via discussion (Figure 1).

The following data were obtained from the studies included in this review: author and date; study sample (sex and mean age); inclusion and exclusion criteria; intervention; follow-up; assessment scales used; and results obtained in the study. This data were compiled in a standard table. The reviewers who selected the articles also independently obtained the data and assessed the methodological quality of the studies. If there were any disagreements, they were resolved via discussion.

### 2.5. Assessment of Methodological Quality

The analysis of the methodological quality of the studies was performed using the PEDro (Physiotherapy Evidence Database) scale [30]. This scale consists of 11 items that can have a ‘yes’ or ‘no’ as a reply. The total range of scores is from 0 to 10 according to a low to excellent methodological quality. The results obtained in the scale were considered as High quality if the score is over 5 (6–8: good, 9–10 excellent), Moderate quality if the score is between 4 and 5 (fair quality study), and Low quality if the score is under 4 (poor quality study). The first item is additional as it is related to external validity and is not used to calculate the score obtained. Therefore, the maximum score is 10. Items 2 to 9 aim to justify if the study has enough internal validity, and items 10 and 11 analyse if the statistical information is appropriate to understand the results. The assessment of the methodological quality of this study was calculated by one reviewer only.

### 2.6. Risk of Bias Analysis

The risk of bias [31] was calculated for each included study, referring to the following types of bias: selection bias; performance bias; detection bias; attrition bias; reporting bias; and other biases. In this assessment, 7 criteria were assessed: 1 = Random sequence generation (selection bias); 2 = Allocation concealment (selection bias); 3 = Blinding of participants and personnel (performance bias); 4 = Blinding of outcome assessment (detection bias); 5 = Incomplete outcome data (attrition bias); 6 = Selective reporting (reporting bias); 7 = Other biases. The risk of bias and the quality of this study were calculated by one reviewer only.

## 3. Results

The literature search was conducted in May 2023. A total of 399 studies were obtained from the search in all databases. The PRISMA flow chart (Figure 1) shows the selection process of the studies. The records that were duplicated were excluded, and 305 records were screened. Finally, 20 studies were included in this review. Table 2 shows the main findings of this review.

Description of the results

The main characteristics of the studies are shown in Table 1. The most relevant aspects are the following:

Sample: A total of 480 patients participated in the included studies. Regarding the number of participants in the different studies, it is worth noting that there was great variability. On the one hand, we observed a sample size of more than 30 patients in six articles [14,37,38,39,40,43,46,47,48] and on the other hand, in four articles [8,33,34,35], the sample size was less than or equal to 7 patients.

We found a higher frequency of men than women [14,26,32,34,35,39,40,42,44,45,47] among the sample. Only four studies [8,37,41,43] had a sample where the female sex prevailed over the male sex but without a significant difference. In the study by Loureiro et al. [33], the sex of the patients was not specified.

Methodology of the studies: All the studies included in this review were clinical trials, but they differed significantly in the methodology applied. Not all the studies had a control group [8,32,33,34,35,44], and those were the studies with smaller samples, so the research was carried out without being able to compare the intervention with other techniques or without a placebo.

In the study conducted by Yen et al. [14], the control group received no treatment at all. In other studies, [37,38] the control group received fall prevention education or the research was based on the comparison of home treatment with virtual reality with supervised treatment in the clinic [39] or routine physical therapy [45,46,47].

In addition, in the study of Calabró et al. [40], we could observe that the procedure was different from the other studies. Although they did not have a control group that did not receive any type of treatment, all the participants completed 20 weeks of conventional physiotherapy, and after three months of rest, they all completed the virtual reality treatment as well.

Virtual reality devices: Regarding the hardware or devices that were used in the different studies, it can be said that there is a predominance of the two most accessible, low-cost devices. On the one hand, there are those studies that used the ‘Nintendo Wii’ [47], the ‘Nintendo Wii + Balance Board’ [33,37,38,39], and on the other hand, there are those that have used the Microsoft Kinect TM [45] in combination with the Kinect Adventures games [34,35,40]. Other studies used different devices, such as the Virtools 3.5 tool [44], a console created by the National Formosa University that applies the treatment through two virtual-reality-based games called Bang Bang Ball and Simulated Board Driving, ‘CAREN’ [42], which is a device composed of a motion capture system and a base platform which is hydraulically driven by the subject’s movements. Moreover, the Interactive video game-based System was used [43], which is a modification of the XaviX Console that applies two games as treatment, one in which the patient performs multidirectional steps and another in which the patient performs steps towards a target. One of the studies [32] used a virtual realisation system created specifically for the study where participants had to process different stimuli and make decisions while walking on the treadmill. One of the most recent studies used the Tymo^®^ system [46], which is a wireless platform for balance and postural control training. The Tymo^®^ system is connected to a screen and provides virtual reality games, adaptable to the functional capacity of the patient. In contrast, two of the analysed studies did not describe the virtual reality device used [41,48].

Type of training and duration of studies: The characteristics of the different treatments vary considerably in the different studies in terms of training volume (number of weeks), frequency, and duration of the sessions. Regarding the volume and frequency of the training, most of the interventions involved about 5–6 weeks of treatment along with two–three sessions per week [14,26,32,37,38,43,44,46]. However, Tunur et al. [8] carried out 3 weeks of treatment but had the highest number of sessions accumulated throughout the week, as they performed daily sessions. Another aspect of this study that can be highlighted is the fact that this training took place in the patient’s own home.

On the other hand, in one study [36], the intervention consisted of 5 weekly sessions over 12 weeks. This period of treatment was similar to the study by Kashif et al. [47], while Hong et al. [48] applied 8 weeks of treatment once a week.

The average duration of each session ranged from 30–60 min in most studies [26,32,34,37,38,39,40,41,42,43,45,46,47]. However, we found two studies that stand out from this average, the one with the longest treatment sessions (75 min) [37] and the one with the shortest (20 min) [34].

Follow-up assessment: All studies conducted a pre-treatment and post-treatment assessment; however, not all of them conducted follow-up evaluations to evaluate whether or not the effectiveness of their intervention was sustained over time. The studies that conducted follow-up carried out the assessments four weeks after the end of the training [14,32,37,38,39,47].

In contrast, Calabró et al. [42] did the follow-up measures at three months, while in the other two investigations [14,40], the follow-up was performed one week after the treatment was completed, and one study did not specify when they established to complete the follow-up assessment [8].

Effects obtained: Several research studies showed that balance improved after virtual reality treatment [26,34,36,37,39,44,45,46]. However, many of them did not perform follow-up evaluations and, thus, did not show evidence of the benefits in the long term.

In the study of Calabró et al. [42], the results were maintained in the long term, even after three months post-intervention. They observed that performing virtual reality training (CAREN) four times a week led to a significant improvement in the gait cycle in terms of duration, speed, length, cadence and step width reduction.

Other research whose results were maintained at the four-week follow-up assessment showed an increase in gait speed, stride length and stride time and an improvement in the 6-m walking test and even in obstacle negotiation [32]. Furthermore, in the study by Kashif et al. [47], the experimental group showed statistically significant improvements in balance at follow-up, with more than 90% of patients showing improvements in this outcome measure.

Yan et al. [26] and Hong et al. [48] used the timed up-and-go test as a measuring tool, which allowed them to prove that the use of virtual reality improved the patient’s functional mobility with consecutive movements (sitting, standing, walking, turning, etc.). However, this improvement was also achieved with conventional home training, as no significant differences were found between virtual reality balance training and conventional home balance training [26]. However, the use of a routine basis treatment combined with virtual reality and Jiao scalp acupuncture made the participants perform the timed up-and-go test in a shorter time than those who received routine basic treatment and virtual reality alone [48].

Most of the results of the studies that compared conventional therapy versus virtual reality showed improvements in gait and balance in both groups. However, the research by Ferraz et al. [40] had three different groups (functional training, exercise bike, and virtual reality training), and only the virtual reality group had a significant improvement in gait speed at the 10-m walking test.

It has also been shown that these improvements in gait occur more effectively in treatments using virtual reality because cognitive and sensory functions are also stimulated. Balance improved notably thanks to the inclusion of integrative function training, which shows the importance of not focusing solely on motor exercises in rehabilitation [44]. Therefore, the use of virtual reality facilitates this treatment approach [44].

The improvement in cardiovascular endurance, which, in turn, influences the improvement in gait, is evidenced in the study carried out by Pompeu et al. [34]. Nevertheless, the maintenance of this improvement at the follow-up was discussed by the authors, as well as the possibility that this improvement was also achieved with conventional physiotherapy.

Regarding balance, we found that most studies used the Berg balance scale to assess this outcome measure. This scale is considered to be the ‘gold standard’ for evaluating functional balance and fall prevention tests that assess the patient’s balance and static abilities. Almost all studies show an improvement in balance in Parkinson’s disease patients after a virtual reality training programme [26,33,36,39,41,42,43,44,46]. However, two investigations found that this improvement in balance was only significant in the experimental group where virtual reality was used [41,43,46].

Methodological quality: The results of the assessment of the methodological quality are shown in Table 3. It should be noted that a negative response does not necessarily mean that the study does not have this characteristic but rather that the requirement was not found in the text even after a thorough review of the article.

The scores obtained in the clinical trials indicated that their methodological quality was fair, with a score of 4–5 [34,35,36,42], and good, with a score of 6–7 [14,26,37,38,39,40,41,45,46,47,48]. Furthermore, we found four articles with poor quality [8,32,33,43], three of which did not have a control group and had a small sample size, and one [43] that, although it had a control group, had a small sample size and presented differences in the baseline data between the groups. Group A had a total of 2 men and 10 women, and group B had 9 men and 3 women, an aspect that may have had a significant influence on the results of the study.

In terms of the study design, it is worth noting that only in three studies [37,38,40] the allocation was concealed. Characteristic 4, or baseline of comparability, was not met by three poorly rated articles [8,32,43]. Another aspect to be taken into account is that none of the articles complied with patient and therapist blinding. In contrast, the follow-up of 85% of the subjects was met in almost all the studies except for three [36,43,44]. The statistical comparability between groups was not met in four articles [8,32,33,35], and the last criterion was met in all the studies except in those carried out by Tunur et al. [8] and Loureiro et al. [33].

## 4. Discussion

The aim of this systematic review was to analyse the efficacy of virtual reality on balance and gait in patients with Parkinson’s disease. Several important aspects are discussed hereafter.

In relation to the effects obtained after the interventions, most of the studies analysed in this review indicated that virtual reality improved gait speed, stride length, balance, gait, and postural control in patients with Parkinson’s disease [14,32,34,37,42,45,46,47]. Furthermore, in the study conducted by Yang et al. [26], the Dynamic Gait Index, Timed up-and-go test and Berg Balance Scale showed significant improvement in both groups, and these changes were maintained during follow-up. Mirelman et al. [32] also achieved improvements in stride length, stride time, gait speed, and obstacle crossing after the intervention with virtual reality, and these significant improvements were maintained at follow-up. Using the CAREN Virtual Reality device, Calabró et al. [42] found that significant improvements in both gait (10-Meter walk test, Timed up-and-Go test and instrumental gait analysis) and balance (Berg Balance Scale) were only obtained at the follow-up assessment in the group that received virtual reality training alone. In the study by Yen et al. [14], there were also improvements after the training and at the subsequent follow-ups. However, there were no significant differences between the virtual reality groups and those with conventional physiotherapy. We believe that in order to know the real effect of virtual reality applications, it is of great importance that all studies follow up on the results over time and not only after the end of the treatment period. Based on these results and those of other research, such as that of Lei et al. [29], virtual reality technology could be considered a rehabilitation approach which is as effective as traditional rehabilitation therapy. Even in outcome measures, such as gait (stride, speed, stride length), balance, and quality of life, the results have shown that virtual reality is better than conventional training.

The role of virtual reality in the rehabilitation of patients with Parkinson’s disease significantly influences the brain’s ability to perceive, process, and integrate information [28]. In this aspect, the study by Pazzaglia et al. [44] showed that after the intervention, there was a significant improvement in balance and gait outcomes in the virtual reality group compared to the control group due to the fact that more cognitive and sensory functions were stimulated than with conventional physiotherapy. Furthermore, in addition to the improvements in the walking ability of Parkinson’s disease patients, progressive increases in muscle strength and sensory integration have been found [38]. A previous systematic review [49] had similar results as virtual reality showed positive effects on balance and gait, as well as other variables, such as activities of daily living function, quality of life, and cognitive function in patients with Parkinson’s disease. They considered that it could be possible that virtual reality provides more comprehensive and accurate motor feedback, which would explain the improvements achieved.

Another benefit that Lei et al. [29] showed about virtual reality is the instantaneous feedback that occurs with these devices, which also improves compliance with rehabilitation training and patient motivation. This coincides with Pompeu et al. [34], who concluded that the main factor that led to improvements in the learning of different motor functions thanks to virtual reality was the presence of continuous visual and auditory feedback provided by the Kinect games throughout the sessions. This aspect was also accounted for by Yang et al. [26], who compared a virtual reality group that focused on visual and audio feedback and a control group that focused on verbal feedback from the therapist. In addition, Feng et al. [41] concluded that the advantage of virtual reality over conventional rehabilitation was that providing continuous feedback improved the patient’s cognitive sensation, increased interest and continuously stimulated the patient’s motivation. As Canning et al. [50] stated, the interaction with the virtual environment and the feedback about performance and success promotes adherence and the success of the treatment.

Motivation and adherence are also influenced by the degree of difficulty and individualisation of the games and tasks performed in the virtual reality intervention. For example, Palacios et al. [35] highlighted the importance of individualisation in the configuration of the parameters of each game, adapting the degrees of difficulty to the ability of each patient. This also occurred in the research conducted by Pompeu et al. [34], where the selection of games was individualised according to the motor and cognitive demands of each patient. Moreover, Domínguez-Ferraz et al. [40] established a gradual progression of the intensity in order to adapt to the different degrees of difficulty of the participants. In this sense, Howard [51] stated that the real impact of virtual reality programmes is achieved through the improvements obtained by patient motivation. In addition, this author supports the idea that the effectiveness of the interventions will depend on the degree of interest that the patients have in these programmes.

Regarding the technological devices that were used in the studies analysed, it is interesting to highlight that most of the virtual reality interventions were carried out through immersive games. The most widely used technology in the studies analysed was the Nintendo Wii Console with the Balance Board accessory. In relation to cost-effectiveness, the Microsoft Kinect TM [34,35,40,45] and the Wii Fit [33,36,37,38,39,47] were the devices that provided the advantages of the use of virtual reality but also reduced the economic cost of the treatment in chronic patients. Therefore, it seems appropriate that those devices should be considered for treatment. The results of the interventions analysed in terms of the devices used coincide with those obtained by other authors who have used virtual reality as a treatment tool, so the use of the Kinect device in the recovery of other pathologies also provides benefits [45,52,53]. In other studies analysed, we can see that different devices were used for the application of virtual reality treatment, such as the balance training system created for the occasion to apply virtual reality treatment in Parkinson’s Disease patients [26] or the Tymo-system [46], obtaining similar benefits to the use of the Kinect device [54].

In most of the studies, the tests were conducted in clinics and specialised centres, and only two studies took place at home [26,39]. The study by Yang et al. [26] showed no significant differences between virtual reality at home compared to conventional home treatment, while Gandolfi et al. [39] concluded that sensory integration performed in the clinic was more effective than virtual reality at home. In contrast, Brachman et al. [45] was the only study that combined one training session a week supervised by a physiotherapist with two sessions performed at home.

In the same way that remote assessment tools such as telemedicine via video calls have been made available in the last years to ensure optimal assessment and treatment monitoring [55], rehabilitation services that can be provided at home without on-site medical supervision should be available, for example, through the use of virtual reality which provides easily accessible and low-cost technological tools [17]. In addition, these instruments can collect a report of the activities performed, allowing for constant feedback and recording of the patient’s progress [23]. Therefore, virtual reality offers the possibility of developing telerehabilitation platforms, where professionals can remotely follow the evolution of the patient from the data recorded during each of the therapy sessions and could apply more personalised interventions to each of the patients [55].

Rehabilitation through virtual reality offers the possibility to carry out the exercises at home, ensuring that the treatment is not interrupted for such reasons as closure of the centre, contagion, difficulty of mobility to the centre, or confinement. On the other hand, we believe that, as rehabilitation can be carried out at home, the patient can do it when he/she feels better (ON phase), allowing for better physical work and greater control of the medication and the disease.

## 5. Limitations of the Study and Further Research

We consider that there is great methodological variability in the research analysed. The inconsistency in the use of assessment tools for the same variable made it difficult to compare results and could lead to different interpretations of the results despite being adequate, current, and validated tools in all the studies included in this review. Therefore, we believe that in future research, it would be necessary to analyse and describe the effects on balance and gait achieved by the application of virtual reality, in addition to an in-depth study of the physiological and psychological effects produced by this type of therapy and the establishment of criteria for inclusion and methodological application that will provide us with the most reliable results [56]. This could be due to the fact that this research is focused on comparing the benefits of these interventions and not on their potential use as a means of assessment.

In terms of methodological quality, the studies included in this review scored between 3 and 7 on the PEDro scale. According to the PEDro interpretation guidelines, if studies scored at least 5 out of 10, they were considered to be of acceptable quality. Studies that scored around 4 did not include blinding of all patients, therapists, and evaluators. Due to the nature of virtual reality interventions, it is very difficult to have triple blinding, as a placebo cannot be used, and the treatment provided is clear to the therapists.

The fact that the studies did not compare virtual reality interventions with each other and only did so with conventional treatments, together with the heterogeneity in the frequency of application of the interventions and number of sessions, means that we cannot conclude which type of virtual reality training is the most appropriate for achieving the greatest benefits in this type of patients. Furthermore, the objective of this study was to analyse the effects of virtual reality in patients with Pakinson’s disease regardless of the type of virtual reality used (immersive or non-immersive), but it would be interesting in future research to analyse this aspect.

Further research is needed to provide better methodological quality and a more solid basis on what effects are achieved by virtual reality, to establish which type of virtual reality training would be the most appropriate and its application in different degrees of the disease, in order to extrapolate the results.

## 6. Implications of the Use of Virtual Reality in Clinical Practice

The results of this systematic review can have positive implications for the clinical practice of professionals working in the rehabilitation field. Virtual reality is a computer-simulated reality that allows the user’s experience of the world he or she perceives to be modified [57].

The studies analysed showed that this technique improves gait and balance in patients with Parkinson’s disease. Furthermore, new virtual reality technologies can provide an engaging and immersive environment for exergaming techniques, maximising goal-oriented training and increasing patients’ self-efficacy during rehabilitation [58]. The results of this review, as well as those from previous ones [28], support that home-based virtual reality can be used as a prolongation to conventional post-clinical rehabilitation programs and help extend the rehabilitation period and favour clinical benefits for patients. Compared to conventional physiotherapy, virtual reality provides the advantage of more personalised training, the possibility of home-based rehabilitation where data can be uploaded in real time and recorded, and allows for greater accessibility, especially in areas with limited access to rehabilitation services [29].

## 7. Conclusions

According to the results of this literature review, virtual-reality-based interventions showed improvements, which are similar to conventional therapy, in the gait variables (gait speed, stride length, decrease in stride width) and balance in patients with Parkinson’s disease. This type of therapy achieves positive results in relation to adherence to treatment, individualisation of the treatment, innovation, motivation, and feedback capacity, as well as great cognitive and sensory stimulation for these patients. Furthermore, thanks to the benefits of virtual therapy, together with the possibility of doing it at home, it allows its application in situations of mobility restrictions. Therefore, virtual reality interventions may be a suitable alternative to the home rehabilitation approach allowing for personalised treatment for these patients.

## Figures and Tables

**Table 1:** Syntaxes of combined descriptors in the scientific database search.

Database	Syntax Adopted
PubMed	‘Parkinson’s Disease AND virtual reality AND gait AND balance’; ‘Parkinson’s disease AND virtual reality AND Physical therapy’; ‘Parkinson’s disease AND virtual reality AND physiotherapy’.
Cochrane	‘Parkinson’s Disease AND virtual reality AND gait AND balance’; ‘Parkinson’s disease AND virtual reality AND Physical therapy’; ‘Parkinson’s disease AND virtual reality AND physiotherapy’.
Dialnet	‘Enfermedad de Parkinson Y realidad virtual Y marcha Y equilibrio’; ‘Enfermedad de Parkinson Y realidad virtual Y Fisioterapia’; ‘Enfermedad de Parkinson Y realidad virtual Y fisioterapia’.
Scopus	‘Parkinson’s Disease AND virtual reality AND gait AND balance’; ‘Parkinson’s disease AND virtual reality AND Physical therapy’; ‘Parkinson’s disease AND virtual reality AND physiotherapy’.
Web of Science	‘Parkinson’s Disease AND virtual reality AND gait AND balance’; ‘Parkinson’s disease AND virtual reality AND Physical therapy’; ‘Parkinson’s disease AND virtual reality AND physiotherapy’.
PsycINFO	‘Parkinson’s Disease AND virtual reality AND gait AND balance’; ‘Parkinson’s disease AND virtual reality AND Physical therapy’; ‘Parkinson’s disease AND virtual reality AND physiotherapy’.
Science Direct	‘Parkinson’s Disease AND virtual reality AND gait AND balance’; ‘Parkinson’s disease AND virtual reality AND Physical therapy’; ‘Parkinson’s disease AND virtual reality AND physiotherapy’.
PEDro (Physiotherapy Evidence Database)	‘Parkinson’s Disease AND virtual reality AND gait AND balance’; ‘Parkinson’s disease AND virtual reality AND Physical therapy’; ‘Parkinson’s disease AND virtual reality AND physiotherapy’.

**Table 2:** Characteristics of the studies.

Authors	Sample, Gender, and Mean Age	Type of Study and Intervention	Treatment and Follow-up Period	Console Type	Assessment Tools	Results
Mirelman et al., 2011 [32]	N = 20 Mean age: 67.1 ± 6.5 Sex: 14 males and 6 males.	RCT Virtual Reality Group: VR simulation designed specifically for this study. In-shoe diodes plus treadmill and screen with virtual environment, plus visual feedback. Comparison was made to a historically active control group of patients with PD who followed a similar protocol of TT but without VR.	Treatment setting not specified. Intervention. 6 weeks of treatment: 18 sessions (3 times a week). Each session lasted 45 min on treadmill with virtual obstacles doing the following: 1. Normal gait: walking at comfortable speed; 2. Walking plus numerical subtraction; 3. Walking plus dodging two obstacles. Assessments. pre-training, post-training, and one month after completion (after 4 weeks) in the ON phase.	The VR simulation was designed specifically for this study. It required the participants to process multiple stimuli simultaneously and challenged them to make decisions about obstacle negotiation in two planes while continuing to walk on the treadmill.	6MWT: assessing resistance GaitRite mat: quantified gait characteristics (stride, stride length, and obstacle clearance), Accelerometer (stride time, quantifying time measurements), Motor part UPDRS (Part III)	Gait speed increased by 8.9%, improved gait length and stride time, and remained on track. Walking plus numerical subtraction, significant improvement in stride length and stride time *p* = 0.016, and improvement in gait variability. 6MWT: improved after training and maintained at follow-up and after training. Obstacle Negotiation: significantly improved speed, gait speed during the 6-m test, and habitual gait speed.
Yen et al., 2011 [14]	N = 42: EG (VR): mean age 70.4 ± 6.5; sex: 2 females and 12 males. CG: mean age 71.6 ± 5.8; sex: 5 females and 9 males, stages II and III	RCT EG (VR): N = 14 EG (CONV treatment): N = 14 CG (no training): N = 14 The 3-dimensional (3D) VR	Treatment setting not specified. Intervention. Training programme of 30 min twice a week for 6 weeks Follow-up evaluations: 7 days post intervention 4 weeks post intervention The first game-based VR experience was called Bang Bang Ball. While the participants played this game, 1 to 5 virtual balls sequentially appeared on a virtual plate that had a hole in the central position, and the virtual plate would move in the same direction as the dynamic balance board. The other VR game, called Simulated Board Driving, included an outdoor simulated environment.	Balanced Training System A dynamic balance board, a 55.88-cm (22-inch) LCD screen, and a personal computer. The dynamic balance board was designed by the Cycling and Health Center, Taichung, Taiwan, and was composed of a tiltable footplate, dual-shaft hinge module, and sensor for interactive training	SOT: 3 sensory systems (visual, somatosensory, and vestibular) in six categories were assessed. Sensory relationships were measured by computerised dynamic posturography (SMART Balance Master).	Pre-training: balance tests with no significant differences. After training: no significant differences between VR and conventional treatment with respect to balance. Better balance in SOT-6 of the VR compared to the conventional treatment (after follow-up this improvement was not significant). Balance training with VR significantly improved the capacity for sensory integration VR group as well as the Conventional treatment group improved in 1 SOT condition. In summary, both VR training and conventional training are beneficial for balance improvement.
Loureiro et al., 2012 [33]	N = 6 mean age 65 ± 13 years old	Pilot study Wii Fit Dynamic Therapy consisted of three planes of movement with stretching and balance exercises using the balance board.	Treatment setting not specified Intervention. Wii activities with 12 sessions of 20 min each performed twice a week in ON phase. Evaluations: start and end of the interventions.	Wii fit plus balance board	Borg scale: to establish the relationship between perceived exertion and external load or stress data. BBS TUG	BBS: Significant change from baseline. TUG: not statistically significant changes when compared to the baseline data.
Pompeu et al., 2014 [34]	N = 7 Sex: 1 female and 6 males. Mean age: 72 ± 9 with PD stage II and III.	Pilot study EG: N = 7, grade II and III	Treatment setting not specified. Intervention. ON Phase, 14 individual training sessions (60 min, 3 times per week), first two sessions with manual and verbal cues. Each game was repeated 3 times/session, 4 min per trial, 3-min break between games and seated game.	Kinect Adventures four games (space pop, 20,000 escapes, reflexion ridge and river rush).	Body function: 6-MWT Balance Evaluation System Test (BESTest) (DGI)	All participants improved their performance in the four games. - 6MWT, BESTest and DGI scores improved after the training. - Cardiopulmonary endurance, balance, mobility, and quality of life improved, although improvements in the 6MWT and DGI were not significant.
Palacios-Navarro et al., 2015 [35]	N = 7 patients with idiopathic PD. Sex: 4 males and 3 females. Mean age: 66.8 ± 3.5	Pilot study EG: N = 7 CG: none	The treatment was carried out at the rehabilitation centre. Intervention: 5-week training (10 h of treatment, 4 sessions/week). The system provided different levels of difficulty; an intermediate and not very demanding level was established in order to perform the exercise. Each training consisted of 30 min of 40 repetitions and was organized into periods, each one corresponding to 40 repetitions of the target. Time between periods was at least 3 min.	Microsoft Kinect TM target. A rehabilitation game based on a low cost device (Microsoft Kinect(TM)) connected to a personal computer.	10 metre walk test (10MWT) at the beginning and at the end (maximum speed). All subjects were taking medication and were in ON PHASE.	Throughout the sessions, there were overall improvements in completion time and in the 10MWT clinical scale *p* = 0.002.
Lee et al., 2015 [36]	N = 20 CG: N = 10 mean age: 70.1 ± 3.3 sex: 5 males and 5 females EG: N = 10 mean age: 68.4 ± 2.9 sex: 5 males and 5 females	Pilot study EG: neurodevelopmental treatment, functional electrical stimulation, plus virtual reality dance exercise (K-Pop Dance Festival) CG: neurodevelopmental treatment and functional electrical stimulation.	Treatment setting not specified. Intervention. 30 min of neurodevelopmental treatment, 15 min of functional electrical stimulation, plus 30 min of dance exercise 5 times a week during 6 weeks. Evaluations. Pre and Post intervention	Wii video games The virtual reality dance exercise used the K-Pop Dance Festival (Nintendo Inc., Japan) game for the Wii (Nintendo Inc., Japan) video game system. Songs liked by patients were selected from the various categories of K-Pop music included in the software. A strap was used to fix the remote control to the hands, and the patients tried to mimic the characters on the TV monitor. When subjects properly mimicked the movement, they felt vibrations from the remote control and heard the word ‘perfect’ broadcast by the TV speaker.	BBS (Balance) Modified Barthel Index (ADL)	Balance: after 6 weeks of treatment balance significantly improved in the EG from 46 to 48.1. In the CG no significant improvement from 45 to 45.4 ADL: changes in ADL had significantly improved in the EG and not in the CG.
Liao et al., 2015 [37]	N = 36 patients stage I, II, III. CG: N = 12 patients mean age: 64.6 ± 8.6 sex: 5 males and 7 females GE (VR): N = 12 patients mean age: 65.1 ± 6.7 sex: 6 males and 6 females GE (traditional exercises): N = 12 Patients mean age: 67.3 ± 7.1 sex: 6 males and 6 females	RT EG (VR): virtual reality-based treatment with Wii Fit EG: traditional exercise group (Stretch, Strength and Balance) CG: falls prevention education	Treatment setting not specified. Intervention. 45 min, followed by 15 min of treadmill training each session. 12 sessions for 6 weeks (2 sessions per week) in ON phase. In the exercise groups, participants received virtual reality-based Wii Fit exercise (VR Wii group) or traditional exercise (TE group) for 45 min, followed by 15 min of treadmill training in each session for a total of 12 sessions over 6 weeks. Participants in the control group received no structured exercise programme. Evaluations. The day before the intervention; day after the intervention and 30 days after the intervention.	Nintendo Wii and balance board.	Primary outcome measures: obstacle crossing performance and dynamic balance (stride length, cross stride speed and vertical clearance and dynamic balance using the LoS-limits of stability test). Secondary outcome measures: SOT; FES-1, TUG	Initial assessment: there were no significant differences between the initial results. Hurdle crossing results: EG (RV): improvements in stride length and stride speed over CG and no differences between EG(VR) and EG (traditional exercise). Balance test and SOT: EG (traditional exercise) and EG (VR): significant improvements over CG. EG(VR): improvements in forward movement in post-training, tracking and lateral movement. TUG: EG (VR) and EG (traditional exercise): significant improvement over GG after training and follow-up.
Liao et al., 2015 [38]	N = 36 patients stage I, II, III. EG (RV): N = 12. Mean age: 65.1 ± 6.7 sex: 6 males and 6 females. EG (traditional exercise): N = 12 patients. Mean age: 67.3 ± 7.1 sex: 6 males and 6 females. CG: N = 12. Mean age: 64.6 ± 8.6 sex: 5 males and 7 females.	RCT EG-1: Wii Fit VR exercise plus 15 min of treadmill. EG-2: traditional exercise plus 15 min treadmill. CG: falls prevention education only.	Treatment setting not specified Intervention. 2 sessions per week during 6 weeks; 45 min of exercise (depending on the group) plus 15 min of treadmill. ON PHASE. Traditional Exercise (TE): This program included 10 min of stretching exercises, 15 min of strengthening exercises, and 20 min of balance exercises in each session. Assessment. pre intervention post intervention one month of follow-up	Nintendo Wii and balance board. Virtual Reality-Based Wii Fit Exercise: The Wii Fit Plus gaming system and Wii Fit balance board (Nintendo Phuten Co, Ltd., Taiwan) were used for VR Wii exercise. The Wii Fit balance board is a novel system that tracks changes in the COP during exercise. A virtual environment was displayed on a screen with a 230 cm width and height in front of the participant. Through avatar technology, images were projected on the screen through a projector. The virtual character provides instantaneous visual and auditory feedback. Participants could imitate the virtual character and adjust their own movements according to feedback	Level walking performance. The GAITRite system (E-T variables: Gait speed and stride length). Functional gait performance Assessment (FGA): ability to modify gait to the task. Muscle strength: hand dynamometer with maximum force for 5 s. SOT: Sensory integration ability	Pre-intervention: there were no significant differences between the groups. Post intervention: Walking skills: VR Wii and Traditional Exercise groups showed significant improvements in gait, length, speed, and FGA compared to the CG. At post intervention and follow-up measurements, there were no significant differences between VR and Traditional exercise groups. Muscle strength: significant differences compared to GC but not between VR Wii and TE. SOT: Significant differences with respect to CG and also significant improvements of EG VR vs. EG traditional exercise at post and follow up.
Yang et al., 2016 [27]	N = 23 patients with idiopathic PD stage II and III. EG: mean age 72.5 ± 8.4 and sex: 4 females and 7 males CG: mean age 75.4 ± 6.3 and sex 5 females and 7 males	RCT EG: N = 11 (Treatment with VR) CG: N = 12 (Conventional treatment)	Treatment setting not specified. Intervention. In ON phase: 12 training sessions of 60 min during 6 weeks at the participants’ home. The VR software had three programs: basic learning; indoor daily tasks; and outdoor daily tasks. The basic learning program helped users familiarise themselves with the VR training system through gaming tasks such as a ball maze. The indoor and outdoor programs simulated daily tasks in indoor and outdoor environments respectively.	Micro-Star International Co. a wireless balance board. The VR balance training system included a 22-inch all-in-one touchscreen computer and a wireless balance board	BBS DGI TUG Unified Parkinson’s rating scale motor score	BBS and DGI were significantly higher than in the initial tests of both VR and CG. Significant improvement in the TUG test in both groups. In summary, both CG and EG with VR showed improvement in balance and gait after training and follow-up. No difference found between VR balance training at home and conventional balance training at home.
Gandolfi et al., 2017 [39]	N = 76 Mean age: 67.45 ± 7.18 Sex: 23 males and 15 females Sex: 28 males and 10 females	RCT EG VR at home: Treatment with Nintendo Wii Tele Wii-Lab with Wii Fit game system and balance board + Skype with the RHB live. EG SI: sensory integration in the clinic	Treatment setting not specified. Intervention. 21 sessions of 50 min each, 3 times a week for 7 weeks in the ON phase. Assessments. Before the intervention, after the intervention and at one month of follow up.	Nintendo Wii with Wii Fit game system and balance board.	BBS ABC 10-MWT for gait speed DGI	Both groups showed a significant overall improvement as measured by ABC scale, 10-MWT, DGI. Improved static and dynamic postural control in EG VR at home. Improvements in mobility and dynamic balance in EG SI. EG SI was more effective than Tele Wii in DGI after training.
Domínguez-Ferraz et al., 2018 [40]	N = 72 (37 males and 25 females) with PD stage 2–2.5–3 G1: N = 22. mean age: 71 ± 4 sex: 16 males and 6 females. G2: N = 20. mean age: 67 ± 4 sex: 11 males and 9 females. G3: N = 20 mean age: 67 ± 1 sex: 10 males and 10 females	A Pilot Single-blinded RCT. G1: functional training (10 activities of 3 min each). G2: exercise on stationary bike (first week 50 HR_max_ and increasing to 75% in the 8th week) G3: training with exergames Kinect Adventures	Treatment setting not specified. Intervention. 8 weeks with 3 sessions per week (50-min sessions). All sessions were performed with a Borg 15 scale (strenuous) In ON phase. Assessments. One week before and 1 week after training.	Xbox 360 Kinect Adventures video game.	Primary outcome measures: - 6MWT (walking ability). - Secondary outcome measures: - 10MWT (walking speed); - SST (muscle strength and power).	All groups showed significant improvements in 6MWT, SST. Only G3 showed significant improvement in gait speed at 10MWT.
Feng et al., 2019 [41]	N = 28 patients with PD grades 2.5 to 4 -EG VR: N = 14. mean age: 67.47 ± 4.79. sex: 8 males and 7 females CG: N = 14. mean age: 66.93 ± 4.64. sex: 6 males and 9 females	RCT EG VR: virtual reality group. Virtual reality technology for balance and gait training. CG: Conventional physiotherapy	Treatment setting not specified Intervention. 45 min sessions of walking and balance training, 5 days a week, for 12 weeks in ON phase. Traditional rehabilitation training group exercise protocol: centre of gravity transfer training carried out in different positions; the force in different directions was given to patients in different contact areas and angles so that patients could control the balance by themselves. Visual, auditory, and orthopaedic mirror feedback methods were used to train the patients to control body posture. Strength training and walking training. Physical therapist in-bed translation training. Exercise both sides of the body while standing or walking. Throwing and catching training. Rhythm training. Experimental group exercise protocol (Game training): warm up; hands and feets touch de ball; hard boating; take the maze; and cool down. Assessment. pre and post intervention	It does not specify the device used to apply the VR treatment.	BBS TUG UPDRS FGA	BBS, TUG and FGA scores were significantly improved in both groups (*p* < 0.05). BBS, TUG and UPDRS were significantly (*p* < 0.05) better in EG. There was no significant difference in UPDRS3 between the pre- and post-rehabilitation data of the control group (*p* < 0.05).
Calabró et al., 2020 [42]	N = 22 patients with PD stage < 3. Sex: 18 males and 4 females Mean age: 66 ± 4	Preliminary study CG: conventional gait training. EG: VR training using CAREN in a personalised way for 40 min.	Treatment setting not specified. Consecutively screening of the outpatients with PD who attended the Behavioral and Robotic Neuro-rehabilitation Laboratory of the IRCCS Neurolesi between August 2017 and October 2018. Intervention. 20 sessions of conventional physiotherapy followed by a 3 months resting period. Then, patients received 20 sessions of CAREN training (40 min of training with 1 or 2 min of rest between exercises) CG: training 4 times a week for 5 weeks (20 sessions) EG: training: 4 times a week for 5 weeks (20 s). Evaluation. At the start of this study, T0, after completion of CG, after completion of CG training T1, and 3 months later T2. After completion of CAREN training T3 and after 3 months of rest T4.	CAREN SYSTEM. Consists of a motion capture system and a base platform driven by hydraulic and mechanical actuators (i.e., a 6–DOF motion platform and built-in instrumented treadmill). The movement of the platform is either driven by the subject’s movements or pre-programmed in synchrony with function curves (that define a specific pathway in the virtual environment). The platform’s movement is synchronized with the visual stimulus (e.g., the platform elevates when the subject arrives at a bump on the screen; the platform tilts accordingly when the road tilts).	Primary outcome measures: BBS; TUG. Secondary outcome measures: MDS-UPDRS II; and III 2. 10 MWT. Instrumental gait analysis with an accelerometer at lumbar level quantifying: (gait cycle length, stride length, gait cycle duration).	Significant improvement in each clinical outcome measure; however, at T2 they returned to baseline results. EG VR: At T4 the improvement in clinical outcome was maintained after the 3-month follow-up. CAREN training slightly shortened the duration of the gait cycle (*p* = 0.04), most evidently the swing phase (*p* = 0.04), increased gait speed (*p* = 0.001) stride length (*p* = 0.02) and percentage of single limb support (*p* < 0.001) and reduced stride width (*p* < 0.001) and cadence (*p* = 0.01).
Yuan et al., 2020 [43]	N = 24 outpatients Group A: N = 12 mean age: 67.8 ± 5.5 sex: 2 males and 10 females Group B: N = 12 mean age 66.5 ± 8.8 sex: 9 males and 3 females	RCT Group A: IVGB treatment in the first 6 weeks plus 6 weeks without IVGB (control week). Group B: first control and then IVGB treatment.	Treatment setting not specified Intervention. Six weeks of IVGB training and then 6 weeks of control. During the intervention phase: training 3 times per week with 30 min training (15 min per task). Assessments. At 6 weeks and at 12 weeks.	IVGB SYSTEM The IVGB system was developed by modifying the XaviX entertainment system. The IVGB exercise program consisted of two tasks: a multi-directional step task and a target-directed stepping task. The IVGB system offers aural and visual feedback in both tasks to increase participants’ attention.	Primary outcome measures: BBS Secondary outcome measures: MSL test. Walking ability and indicator of mobility function and risk of falls.	- Initial score: MMSE and the percentage of women were significantly lower in Group B than in Group A. - Post intervention: BBS: Group B at week 12 higher score than initially and at week 6. MSL test: Group B higher at week 12 than initially. BBS and two MSL scales were significantly different between the two groups at 6 weeks. A 6-week IVGB training significantly improved balance ability and MSL in left, right and backward directions in group B patients. IVGB: virtual reality tasks improved motor coordination and the ability to stand on one leg.
Pazzaglia et al., 2020 [44]	N = 51. 16 females and 35 males. EG: Sex: 18 males and 7 females with mean age: 72 ± 7. CG: Sex: 17 males and 9 females. Mean age: 70 ± 10	RCT EG: N = 25 RHB with VR CG: N = 26 conventional RHB ON phase	Treatment setting not specified. Duration of the programme: 6 consecutive weeks. Each session 40 min 3 times per week.	Virtools 3.5 Using Virtools 3.5 were developed by National Formosa University and authorized by the Cycling and Health Centre.	BBS: to measure balance. Secondary outcome measures: DGI: to evaluate the ability to adapt gait to complex walking tasks. DAHS questionnaire: to measure performance of the upper limb. SF-36 questionnaire: to evaluate quality of life.	Patients in the EG showed better balance and gait outcomes BBS *p* = 0.003: - Pre-intervention: EG: 45.6 (7.9) and CG: 47.3 (7.6). - Post-intervention: EG 49.2 (8.19 and CG: 48.1 (7.2) - Significant difference: EG: 3.6 and CG: 0, - DGI: - Pre-intervention: EG: 18.7 (4.7) and CG 19.1 (2.9). - Post-intervention: EG: 20.2 (4.2) and CG: 19 (3.9) - Significant difference: EG: 1.6 and CG: −0.2.
Tunur et al., 2020 [8]	N = 7 patients with PD Mean age: 69 years Sex: 4 females and 3 males.	Pilot study EG: MTG demo plus use of Google Glass	Treatment setting not specified. Intervention. 3 weeks of treatment; 20-min demonstration of VR use; Completion of the four MTG modules supervised by the researchers; Home use for 3 weeks minimum 3 modules per day. Evaluation Baseline; Post-test; Follow.	Google Glass All applications and features normally found in Google Glass were removed for this study to prevent personal information from being collected. The Google Glass were defaulted to an offline environment that only contained the MTG modules. The MTG was voice-activated using the prompt ‘OK Glass’, followed by choosing the preferred MTG module from the list of four MTG modules: Warm Me Up; Balance Me; Unfreeze Me; Walk with Me. The participants could use voice-activated commands, or swipe and tap the control bar to navigate through the menu. Each of the first three modules have three or four different movement variations, averaging approximately 45 s per video.	- Mini-Best: to assess balance and functional mobility. - Posture of the most affected leg. - Dual task: walking and talking. TUG plus counting backwards.	- No alterations in balance or mobility scores were observed. - Significant improvement in dual-task cost after 3 weeks of use.
Brachman et al., 2021 [45]	N = 24 patients with PD EG: 12 Mean age 69.5 ± 7.2 Sex: 8 males and 4 females. EC: 12 Mean age 65.3 ± 9.2 Sex: 7 males and 5 females	RCT EG: was trained with a custom made exergaming balance-based training system. CG: a conventional balance training.	Treatment setting: A Virtual Reality scenario was projected on the 65-inch screen situated 2 m away in front of the participant. Intervention: 12 balance-based exergaming training sessions; 3 sessions per week using the exergaming balance training system. Each training lasted for 30 min. Participants in the control group received 12 training sessions (3 sessions per week) of the conventional balance rehabilitation. Evaluation Before intervention (pre-training); The day after the completion of a training program (post-training).	The VR system included two integrated devices, a Kinect sensor system and a custom made force platform. Patients were introduced inside the video game as an avatar character which provided instantaneous visual feedback about participants’ performance. In each training session patients practiced maintaining static posture, leaning in different directions, dynamic weight shifting, gait initiation, step making and trunk rotation.	All measurements were performed on a force platform, which was part of a VR-based balance system. Postural stability: Quiet Standing Eyes Open. Quiet Standing Eyes Closed. Dynamic balance: FBT LOS	After training, participants in both groups showed significantly better results in static balance performance. However, only exergaming training significantly improved LOS performance (higher values of Range of forward lean (*p* = 0.039, dz = 0.67) and leaning rate (*p* = 0.007, dz = 0.96). Also FBT test improved significantly only in experimental group (decrease in time to target hit (*p* = 0.02, dz = 0.76) and significant increase in average COP velocity (*p* = 0.008, dz = 0.93).
Maranesi et al., 2022 [46]	N = 30 patients with PD EG: 16 Mean age: 72.7 ± 6.3 Sex: 6 males and 10 females CG: 14 Mean age: 75.1 ± 5.4 Sex: 9 males and 5 females	RCT preliminary result. EG: technological Rehabilitation. CG: traditional rehabilitation.	A 10-sessions training was conducted, divided into 2 sessions per week, for 5 weeks. CG performed traditional therapy sessions lasting 50 min each. EG: The technological intervention group carried out 30 min of traditional therapy and 20 min of treatment. Traditional rehabilitation treatments, consisting of breathing and relaxation, task-oriented exercise to improve strength and to reduce limitations in the activities of daily living, walking with cues to reduce gait deficit, stretching to relieve muscle and joint stiffness, static and dynamic balance training to reduce postural control impairments, flexibility exercises to improve the range of motion of different joint, unilateral and contralateral coordination exercises performed in bed and while standing, involving 4 limbs. Evaluation Baseline; Post-test.	Tymo^®^ system: a wireless platform that provides non-immersive virtual reality exergames, which can be adapted to each patient according to the functional capacity, in order to improve balance and postural control; the system offers a number of therapy games from Verena Schweizer’s neuro-training	CDR PIADS BI SF-12 FES-I Gait and balance performance on Tinetti’s POMA	There was an improvement in balance at the end of treatments in both groups (CG: 12.4 ± 0.7 vs. 13.5 ± 0.8, *p* = 0.017; TG: 13.8 ± 0.5 vs. 14.7 ± 0.4, *p* = 0.004). The overall risk of falls was significantly reduced only in the experimental technology rehabilitation group (POMA Total: 24.6 ± 0.9 vs. 25.9 ± 0.7, *p* = 0.010). All POMA scores differed statistically significantly in the EG, highlighting the improvement not only in balance but also in gait characteristics (9.7 ± 0.8 vs. 11.4 ± 0.2, *p* = 0.003). There was also an improvement in the psychological sphere in the EG, measured through the MSC-(17.1 ± 0.4 vs. 16.5 ± 0.4, *p* = 0.034).
Kashif et al., 2022 [47]	N = 44 patients with idiopathic PD EG; 22 Mean age: 63.86 ± 4.57 Sex: 13 male and 9 female CG: 22 Mean age: 62.32 ± 4.61 Sex: 12 male and 10 female	RCT EG: routine physical therapy along with VR and MI techniques CG: routine physical therapy (warm-up, stretching, strengthening, and relaxation exercises, limb coordination exercises, trunk, neck, and gait training),	For safety purposes, the patients stood inside parallel bars on the Wii Fit board with their shoes of EG received 60-min sessions: 40-minroutine physical therapy as in EC; 10–15 min of VR; and 5–10 min of MI techniques. Every other day (three days a week) for 12 weeks, CG received 40-min sessions and 20 min of walking and cycling, with a short rest period every other day (three days a week) for 12 weeks. Evaluation: - Baseline; - 6th Week 12th Week; - Follow: 16th week.	The VR system consisted of a wall-mounted display, a Wii box, a Wii remote, and a Wii Fit board. The patients were instructed to stand on Wii Fit board while interacting with the VR system and playing the selected games. The games were of motor functionality, balance and ADL. The last 5–10 min of the session comprised the MI. Consisted of watching the recorded videos, analyzing the differences in both videos, and the differences in both videos. In the next step, they were instructed to relax and concentrate on their calm breathing patterns. To finish by performing the activities, they were given verbal commands whenever necessary.	Motor Function UPDRS-part III. Balance confidence ABCS. BBS ADL UPDRS-part II	The experimental group showed a more significant improvement in motor function than the control group on UPDRS part III, with 32.45 ± 3.98 vs. 31.86 ± 4.62 before and 15.05 ± 7.16 vs. 25.52 ± 7.36 at 12 weeks, and *p*-value < 0.001. At 12 weeks, the BBS scores of the experimental group improved from 38.95 ± 3.23 to 51.36 ± 2.83, with a *p*-value < 0.001. At 12 weeks, the experimental group’s balance confidence improved significantly, from 59.26 ± 5.87 to 81.01 ± 6.14, with a *p*-value < 0.001. The ADL scores of the experimental group also improved, going from 22.00 ± 4.64 to 13.07 ± 4.005 after 12 weeks, with a *p*-value < 0.001.
Hong et al., 2022 [48]	N = 52 patients with PD EG: 26 CG: 26	CG: Routine basic treatment and VR rehabilitation training. EG: Same as control group but added Jiao scalp acupuncture. Scalp points included movement area, balance area, and dance tremor control area.	Both groups were treated once a day, 5 times a week, for a total of 8 weeks.	It does not specify the device used to apply the VR treatment.	The gait parameters (step distance, step width, step speed, and step frequency). Timed ‘up-and-go’ test. Unified Parkinson’s disease rating scale part Ⅲ (UPDRS-Ⅲ)	Four weeks into treatment: - The gait parameters of both groups improved, the TUGT time was shortened, and the UPDRS-Ⅲ scores were reduced (*p* < 0.01, *p* < 0.05); - The step distance in the observation group was better than in the control group, and the UPDRS-Ⅲ score in the observation group was lower than in the control group (*p* < 0.05). Eight weeks into treatment: - The distance and speed of steps in the observation group were better than in the control group; the TUGT time in the observation group was shorter than in the control group, and the UPDRS-Ⅲ score in the control group observation was lower than in the control group (*p* < 0.05, *p* < 0.01).

Note: RCT: Randomized Controlled Trial; RT: Randomized Trial; PD: Parkinson’s disease; EG: Experimental group; CG: Control group; G: Group; MWT: Meter walk test; RHB: Rehabilitation; VR: Virtual Reality; BBS: Berg Balance Scale; DGI: Dynamic Gait Index; CONV: Conventional; SOT: Sensory organisation test; BESTest: Balance Evaluation System Test; TUG: Timed Up-and-Go test; FES: Falling Effectiveness Scale; LoS: Limits of stability; UPDRS: Unified Parkinson’s Disease Rating Scale; SI: Sensory Integration; ABC: Activity Specific Balance Confidence Scale; HR_max_: Maximum Heart Rate; SST: Sit-to-stand test; FGA: Functional gait assessment; MDS: Movement Disorders Society; IVGB: Interactive video game-based; MSL: Maximum stride length; DAHS: Disabilities of the Arm, Shoulder, and Hand questionnaire, SF-36: Short Form 36 health survey questionnaire: COP: Centre of pressure; MI: Motor imagery; ADL: Activities of Daily livings. TT: Treadmill training; TE: Traditional Exercise; MTG: Moving Through Glass; FBT: Functional Balance Test; CDR; Clinical Dementia Rating Scale; PIADS: The psychosocial impact of assistive devices scale; BI: Barthel Index; POMA: Performance Oriented Mobility Assessment.

**Table 3:** Physiotherapy Evidence Database (PEDro) scale.

Study	Criteria											Score	Result
	1	2	3	4	5	6	7	8	9	10	11		
Mirelman et al., 2011 [32]	N	N	N	N	N	N	N	Y	Y	N	Y	3	POOR
Yen et al., 2011 [14]	N	Y	N	Y	N	N	Y	Y	Y	Y	Y	7	GOOD
Loureiro et al., 2012 [33]	Y	N	N	Y	N	N	N	Y	N	Y	N	3	POOR
Pompeu et al., 2014 [34]	Y	N	N	Y	N	N	Y	Y	Y	N	Y	5	FAIR
Palacios-Navarro et al., 2015 [35]	N	N	N	Y	N	N	N	Y	Y	N	Y	4	FAIR
Lee et al., 2015 [36]	N	Y	N	Y	N	N	N	N	N	Y	Y	4	FAIR
Liao et al., 2015 [37]	Y	Y	Y	Y	N	N	Y	Y	N	Y	Y	7	GOOD
Liao et al., 2015 [38]	Y	Y	Y	Y	N	N	Y	Y	N	Y	Y	7	GOOD
Yang et al., 2016 [26]	Y	Y	N	Y	N	N	Y	Y	Y	Y	Y	7	GOOD
Gandolfi et al., 2017 [39]	Y	Y	N	Y	N	N	Y	Y	N	Y	Y	6	GOOD
Domínguez-Ferraz et al., 2018 [40]	Y	Y	Y	Y	N	N	Y	Y	N	Y	Y	7	GOOD
Feng et al., 2019 [41]	Y	Y	N	Y	N	N	Y	Y	Y	Y	Y	7	GOOD
Calabró et al., 2019 [42]	Y	N	N	Y	N	N	N	Y	Y	Y	Y	5	FAIR
Yuan et al., 2020 [43]	Y	Y	N	N	N	N	N	N	N	V	Y	3	POOR
Pazzaglia et al., 2020 [44]	N	Y	N	Y	N	N	Y	N	N	Y	Y	5	FAIR
Tunur et al., 2020 [8]	Y	N	N	N	N	N	Y	Y	Y	N	N	3	POOR
Brachman et al., 2021, [45]	Y	Y	N	Y	N	N	Y	Y	Y	Y	Y	7	GOOD
Maranesi et al., 2022 [46]	Y	Y	N	Y	N	N	Y	Y	Y	Y	Y	7	GOOD
Kashif et al., 2022 [47]	Y	Y	N	Y	N	N	Y	Y	Y	Y	Y	7	GOOD
Hong et al., 2022 [48]	Y	Y	N	Y	N	N	Y	Y	Y	Y	Y	7	GOOD

N: Did not meet the criteria; Y: Met the criteria. 1. Eligibility criteria were specified; 2. Random allocation; 3. Concealed allocation; 4. Similar groups at baseline; 5. Blinding of all subjects; 6. Blinding of all therapists; 7. Blinding of all assessors; 8. Follow up of more than 85% of the subjects; 9. Intention to treat analysis; 10. Between-group statistical comparisons; 11. Point measures and measures of variability for at least one key outcome are given.

## Data Availability

Data is available upon reasonable request to the authors.

## References

[1] Balestrino R., Schapira A.H.V. (2020). Parkinson disease. Eur. J. Neurol..

[2] Pringsheim T., Jette N., Frolkis A., Steeves T.D. (2014). The prevalence of Parkinson’s disease: A systematic review and meta-analysis. Mov. Disord..

[3] Li J., Jin M., Wang L., Qin B., Wang K. (2015). MDS clinical diagnostic criteria for Parkinson’s disease. Mov. Disord..

[4] Micheli F., Fernández Pardal M. (2011). Neurología. Neurología.

[5] Chouza M., Raposo I., Fernández R., González L., Martínez A., Fernández M.A. (2001). Protocolo de Fisioterapia en el paciente parkinsoniano. Fisioterapia.

[6] Moreno C.L., Cleves C.C. (2019). Acta Neurológica colombiana Consenso de la asociación Colombiana de neurología sobre enfermedad de Parkinson. Acta Neurol. Colomb..

[7] Kulisevsky J. Enfermedad de Parkinson: Guía Terapéutica de la Sociedad Catalana de Neurología. https://docplayer.es/7259662-Guia-terapeutica-de-la-sociedad-catalana-de-neurologia.html.

[8] Tunur T., DeBlois A., Yates E., Rickford K., Columna L.A. (2020). Augmented reality-based dance intervention for individuals with Parkinson’s disease: A pilot study. Disabil. Health J..

[9] Earhart G.M., Dibble L.E., Ellis T., Nieuwboer A. (2012). Rehabilitation and Parkinson’s disease. Parkinsons Dis..

[10] Tomlinson C.L., Herd C.P., Clarke C.E., Meek C., Patel S., Stowe R., Deane K.H., Shah L., Sackley C.M., Wheatley K. (2014). Physiotherapy for parkinson’s disease: A comparison of techniques. Cochrane Database Syst. Rev..

[11] Tomlinson C.L., Patel S., Meek C., Herd C.P., Clarke C.E., Stowe R., Shah L., Sackley C., Deane K.H., Wheatley K. (2012). Physiotherapy intervention in Parkinson’s disease: Systematic review and meta-analysis. BMJ.

[12] Morales S., Elizagaray I., Yepes Ó., de la Puente L., Gil A. (2018). Effectiveness of virtual immersion programmes in patients with parkinson’s disease. A Syst. Rev. Rev. Neurol..

[13] Viñas S., Sobrido M. (2016). Realidad virtual con fines terapéuticos en pacientes con ictus: Revisión sistemática. Neurologia.

[14] Yen C.Y., Lin K.H., Hu M.H., Wu R.M., Lu T.W., Lin C.H. (2011). Effects of virtual reality-augmented balance training on sensory organization and attentional demand for postural control in people with Parkinson disease: A randomized controlled trial. Phys. Ther..

[15] Domínguez P., Moral J.A., Casado E., Salazar A., Lucena D. (2019). Effects of virtual reality on balance and gait in stroke: A systematic review and meta-analysis. Rev. Neurol..

[16] Peñasco B., De Los Reyes A., Gil Á., Bernal A., Pérez B., De La Peña A.I. (2010). Application of virtual reality in the motor aspects of neurorehabilitation. Rev. Neurol..

[17] Robles V. (2018). Virtual reality as a tool in physiotherapy: Fiction or reality?. Fisioterapia.

[18] Ferreira dos Santos L., Christ O., Mate K., Schmidt H., Krüger J., Dohle C. (2016). Movement visualisation in virtual reality rehabilitation of the lower limb: A systematic review. BioMed. Eng. OnLine.

[19] Vavoda J.T. (2007). Virtual Humanoids and Presence in Virtual Environments. Ph.D. Thesis.

[20] Bayón M., Martínez J. (2010). Rehabilitación Del Ictus Mediante Realidad Virtual. Rehabilitación.

[21] Iacoboni M., Woods R.P., Brass M., Bekkering H., Mazziotta J.C., Rizzolatti G. (1999). Cortical mechanism of human imitations. Science.

[22] Kim A., Darakjian N., Finley J.M. (2017). Walking in fully immersive virtual environments: An evaluation of potential adverse effects in older adults and individuals with Parkinson’s disease. J. Neuroeng. Rehabil..

[23] Torres-Narváez M., Sánchez-Romero J., Pérez-Viatela A., Betancu E., Villamil-Ballesteros J., Valero-Sánchez K. (2018). Entrenamiento motor en el continuo de la realidad a la virtualidad. Fac. Med..

[24] Schultheis M.T., Rizzo A.A. (2001). The application of virtual reality technology in rehabilitation. Rehabil. Psychol..

[25] Bailenson J., Patel K., Nielsen A., Bajscy R., Jung S.-H., Kurillo G. (2008). The Effect of Interactivity on Learning Physical Actions in Virtual Reality. Med. Psychol..

[26] Yang W.C., Wang H.K., Wu R.M., Lo C.S., Lin K.H. (2016). Home-based virtual reality balance training and conventional balance training in Parkinson’s disease: A randomized controlled trial. J. Formos. Med. Assoc..

[27] Hutton B., Catalá F., Moher D. (2016). La extensión de la declaración PRISMA para revisiones sistemáticas que incorporan metaanálisis en red: PRISMA-NMA. Med. Clin..

[28] Dockx K., Bekkers E.M., Van den Bergh V., Ginis P., Rochester L., Hausdorff J.M., Mirelman A., Nieuwboer A. (2016). Virtual reality for rehabilitation in Parkinson’s disease. Cochrane Database Syst. Rev..

[29] Lei C., Sunzi K., Dai F., Liu X., Wang Y., Zhang B., He L., Ju M. (2019). Effects of virtual reality rehabilitation training on gait and balance in patients with Parkinson’s disease: A systematic review. PloS ONE.

[30] PEDro Scale (Escala PEDro). http://www.pedro.org.au/spanish/downloads/pedro-scale/.

[31] Higgins J.P.T., Altman D.G., Sterne J.A.C., Higgins J.P.T., Green S. (2011). Assessing risk of bias in included studies. Cochrane Handbook of Systematic Review of Interventions, Version 510.

[32] Mirelman A., Maidan I., Herman T., Deutsch J.E., Giladi N., Hausdorff J.M. (2011). Virtual reality for gait training: Can it induce motor learning to enhance complex walking and reduce fall risk in patients with Parkinson’s disease?. J. Gerontol. A Biol. Sci. Med. Sci..

[33] Loureiro A.P.C., Ribas C.G., Zotz T.G.G., Chen R., Ribas F. (2012). Feasibility of virtual therapy in rehabilitation of Parkinson’s disease patients: Pilot study. Fisioter. Mov..

[34] Pompeu J.E., Arduini L.A., Botelho A.R., Fonseca M.B.F., Pompeu S.M.A.A., Torriani-Pasin C., Deutsch J.E. (2014). Feasibility, safety and outcomes of playing Kinect Adventures!TM for people with Parkinson’s disease: A pilot study. Physiotherapy.

[35] Palacios G., García I., Ramos P. (2015). A Kinect-Based System for Lower Limb Rehabilitation in Parkinson’s Disease Patients: A Pilot Study. J. Med. Syst..

[36] Lee N.Y., Lee D.K., Song H.S. (2015). Effect of virtual reality dance exercise on the balance, activities of daily living, And depressive disorder status of Parkinson’s disease patients. J. Phys. Ther. Sci..

[37] Liao Y.Y., Yang Y.R., Cheng S.J., Wu Y.R., Fuh J.L., Wang R.Y. (2015). Virtual Reality-Based Training to Improve Obstacle-Crossing Performance and Dynamic Balance in Patients With Parkinson’s Disease. Neurorehabilit. Neural Repair..

[38] Liao Y.Y., Yang Y.R., Wu Y.R., Wang R.Y. (2015). Virtual Reality-Based Wii Fit Training in Improving Muscle Strength, Sensory Integration Ability, and Walking Abilities in Patients with Parkinson’s Disease: A Randomized Control Trial. Int. J. Gerontol..

[39] Gandolfi M., Geroin C., Dimitrova E., Boldrini P., Waldner A., Bonadiman S., Picelli A., Regazzo S., Stirbu E., Primon D. (2017). Virtual Reality Telerehabilitation for Postural Instability in Parkinson’s Disease: A Multicenter, Single-Blind, Randomized, Controlled Trial. Biomed. Res. Int..

[40] Ferraz D.D., Trippo K.V., Duarte G.P., Neto M.G., Bernardes K.O., Filho J.O. (2018). The Effects of Functional Training, Bicycle Exercise, and Exergaming on Walking Capacity of Elderly Patients With Parkinson Disease: A Pilot Randomized Controlled Single-blinded Trial. Arch. Phys. Med. Rehabil..

[41] Feng H., Li C., Liu J., Wang L., Ma J., Li G., Gan L., Shang X., Wu Z. (2019). Virtual reality rehabilitation versus conventional physical therapy for improving balance and gait in parkinson’s disease patients: A randomized controlled trial. Med. Sci. Monit..

[42] Calabrò R.S., Naro A., Cimino V., Buda A., Paladina G., Di Lorenzo G., Manuli A., Milardi D., Bramanti P., Bramanti A. (2020). Improving motor performance in Parkinson’s disease: A preliminary study on the promising use of the computer assisted virtual reality environment (CAREN). Neurol. Sci..

[43] Yuan R.Y., Chen S.C., Peng C.W., Lin Y.N., Chang Y.T., Lai C.H. (2020). Effects of interactive video-game-based exercise on balance in older adults with mild-to-moderate Parkinson’s disease. J. Neuroeng. Rehabil..

[44] Pazzaglia C., Imbimbo I., Tranchita E., Minganti C., Ricciardi D., Monaco R.L., Parisi A., Padua L. (2020). Comparison of virtual reality rehabilitation and conventional rehabilitation in Parkinson’s disease: A randomised controlled trial. Physiotherapy.

[45] Brachman A., Marszałek W., Kamieniarz A., Michalska J., Pawłowski M., Juras G. (2021). Biomechanical measures of balance after balance-based exergaming training dedicated for patients with Parkinson’s disease. Gait Posture.

[46] Maranesi E., Casoni E., Baldoni R., Barboni I., Rinaldi N., Tramontana B., Amabili G., Benadduci M., Barbarossa F., Luzi R. (2022). The Effect of Non-Immersive Virtual Reality Exergames versus Traditional Physiotherapy in Parkinson’s Disease Older Patients: Preliminary Results from a Randomized-Controlled Trial. Int. J. Environ. Res. Public Health.

[47] Kashif M., Ahmad A., Bandpei M.A.M., Gilani S.A., Hanif A., Iram H. (2022). Combined effects of virtual reality techniques and motor imagery on balance, motor function and activities of daily living in patients with Parkinson’s disease: A randomized controlled trial. BMC Geriatr..

[48] Hong Z.M., Qiu J.F., Zhang S.Q., Wang Y.B., He K.L., Ma R.J. (2022). Jiao’s scalp acupuncture combined with virtual reality rehabilitation training for motor dysfunction in patients with Parkinson’s disease: A randomized controlled trial. Zhongguo Zhen Jiu.

[49] Canning C.G., Allen N.E., Nackaerts E., Paul S.S., Nieuwboer A., Gilat M. (2020). Virtual reality in research and rehabilitation of gait and balance in Parkinson disease. Nat. Rev. Neurol..

[50] Howard M.C. (2017). A meta-analysis and systematic literature review of virtual reality rehabilitation programs. Comput. Human. Behav..

[51] Ikbali Afsar S., Mirzayev I., Umit Yemisci O., Cosar Saracgil S.N. (2018). Virtual Reality in Upper Extremity Rehabilitation of Stroke Patients: A Randomized Controlled Trial. J. Stroke Cerebrovasc. Dis..

[52] Lloréns R., Noé E., Colomer C., Alcañiz M. (2015). Effectiveness, usability, and cost-benefit of a virtual reality-based telerehabilitation program for balance recovery after stroke: A randomized controlled trial. Arch. Phys. Med. Rehabil..

[53] Sá M.J. (2014). Exercise therapy and multiple sclerosis: A systematic review. J. Neurol..

[54] Truijen S., Abdullahi A., Bijsterbosch D., van Zoest E., Conijn M., Wang Y., Struyf N., Saeys W. (2022). Effect of home-based virtual reality training and telerehabilitation on balance in individuals with Parkinson disease, multiple sclerosis, and stroke: A systematic review and meta-analysis. Neurol. Sci..

[55] Peñasco-Martín B., De los Reyes-Guzmán A., Gil-Agudo A., Bernal-Sahún A., Pérez-Aguilar B., De la Peña-González A.I. (2010). Aplicación de la realidad virtual en los aspectos motores de la neurorrehabilitación. Rev. Neurol..

[56] Abbas R.L., Cooreman D., Al Sultan H., El Nayal M., Saab I.M., El Khatib A. (2021). The Effect of Adding Virtual Reality Training on Traditional Exercise Program on Balance and Gait in Unilateral, Traumatic Lower Limb Amputee. Games Health J..

[57] Tack C. (2021). Virtual reality and chronic low back pain. Disabil. Rehabil. Assist. Technol..

[58] Bond S., Laddu D.R., Ozemek C., Lavie C.J., Arena R. (2021). Exergaming and Virtual Reality for Health: Implications for Cardiac Rehabilitation. Curr. Probl. Cardiol..

